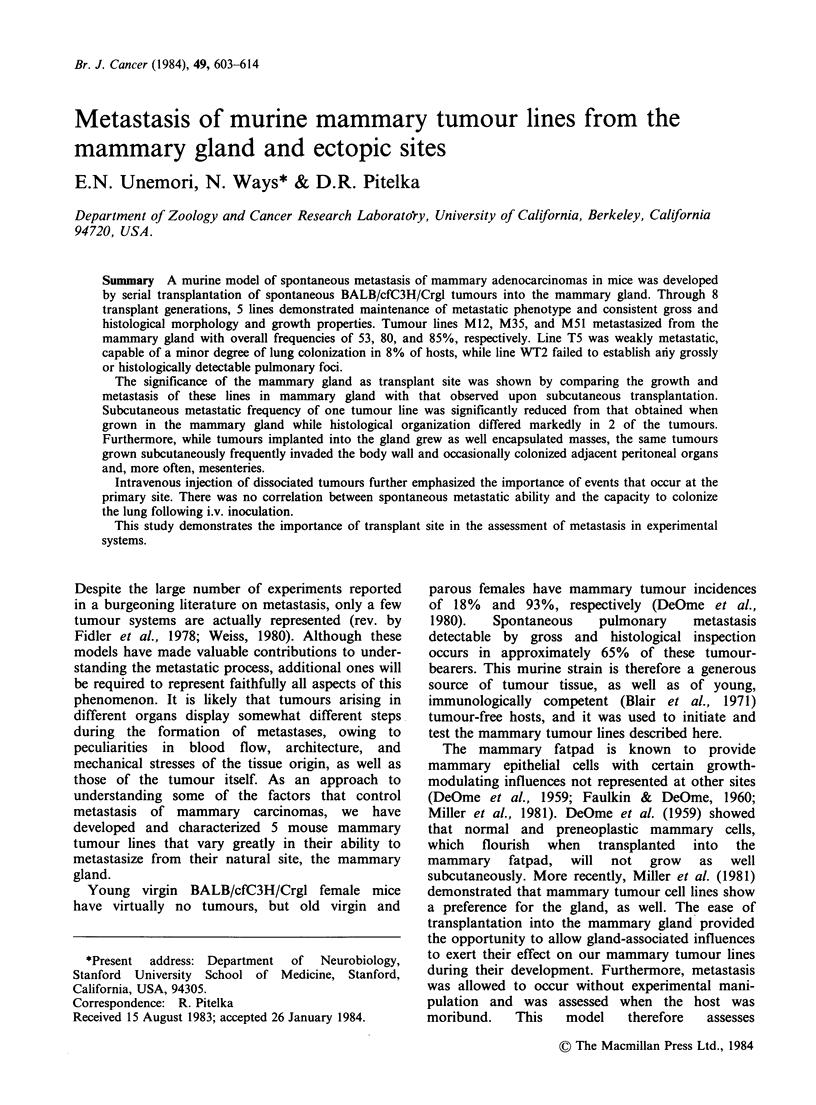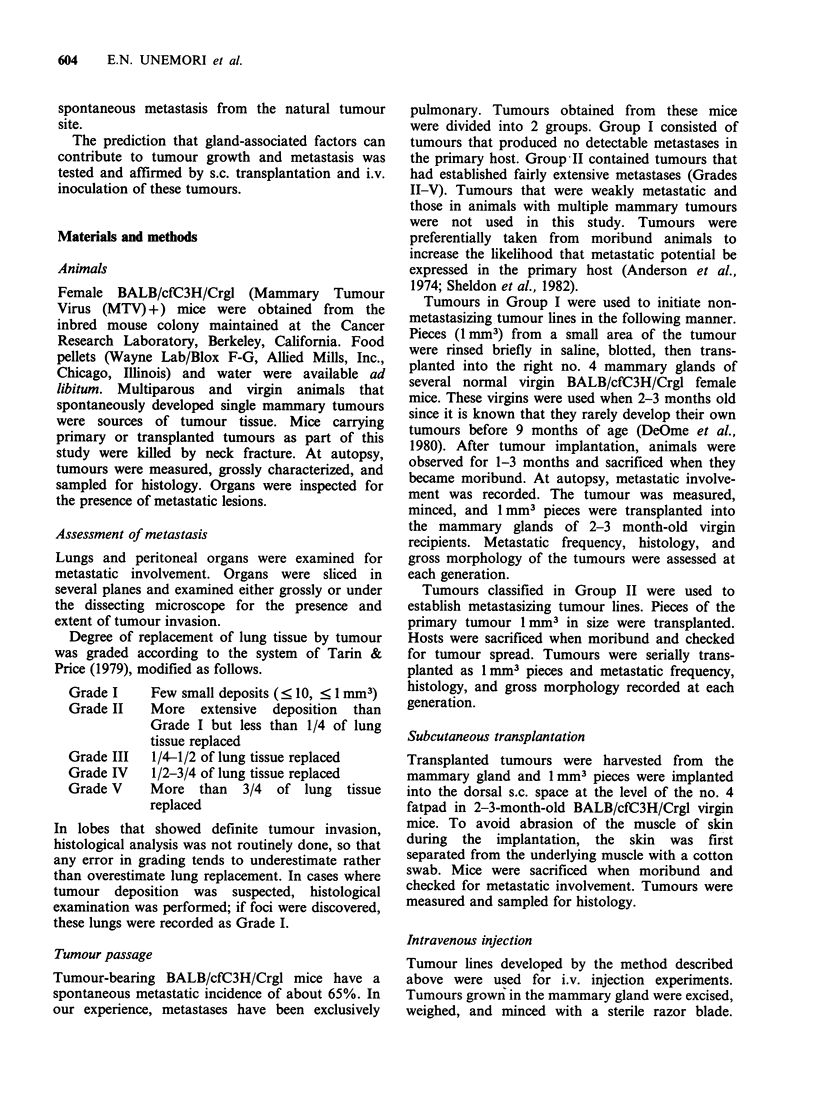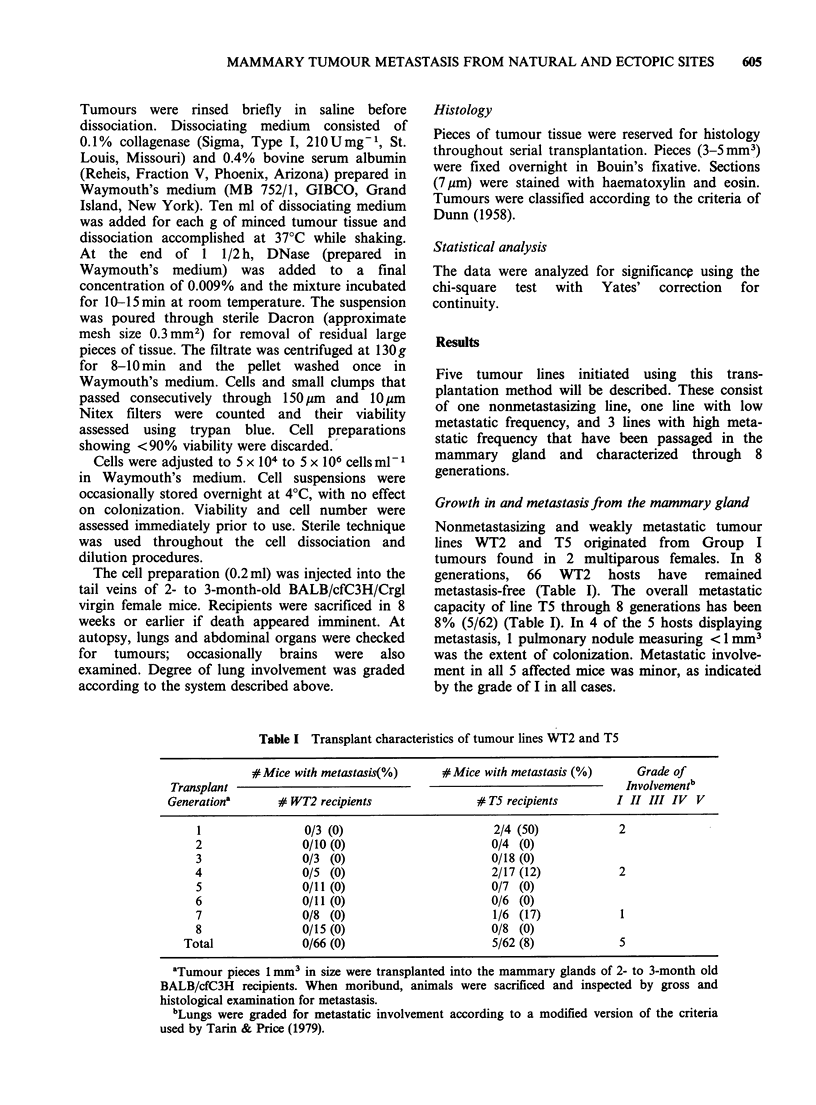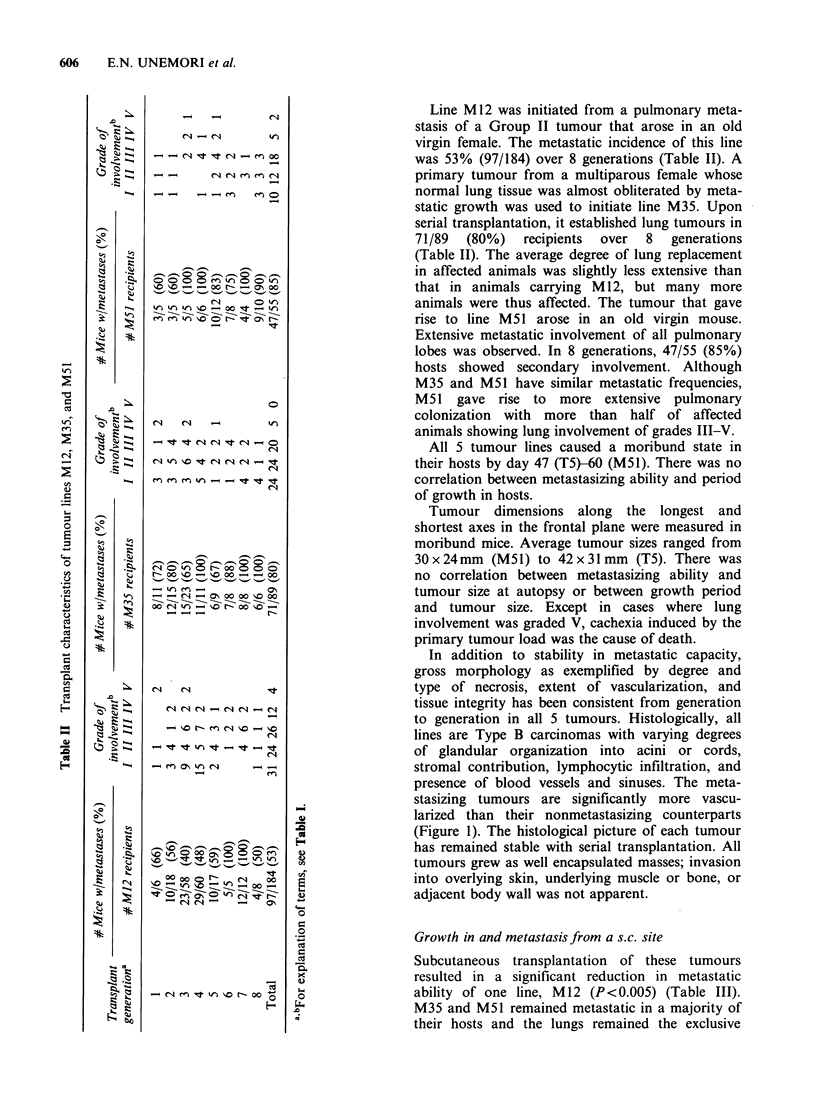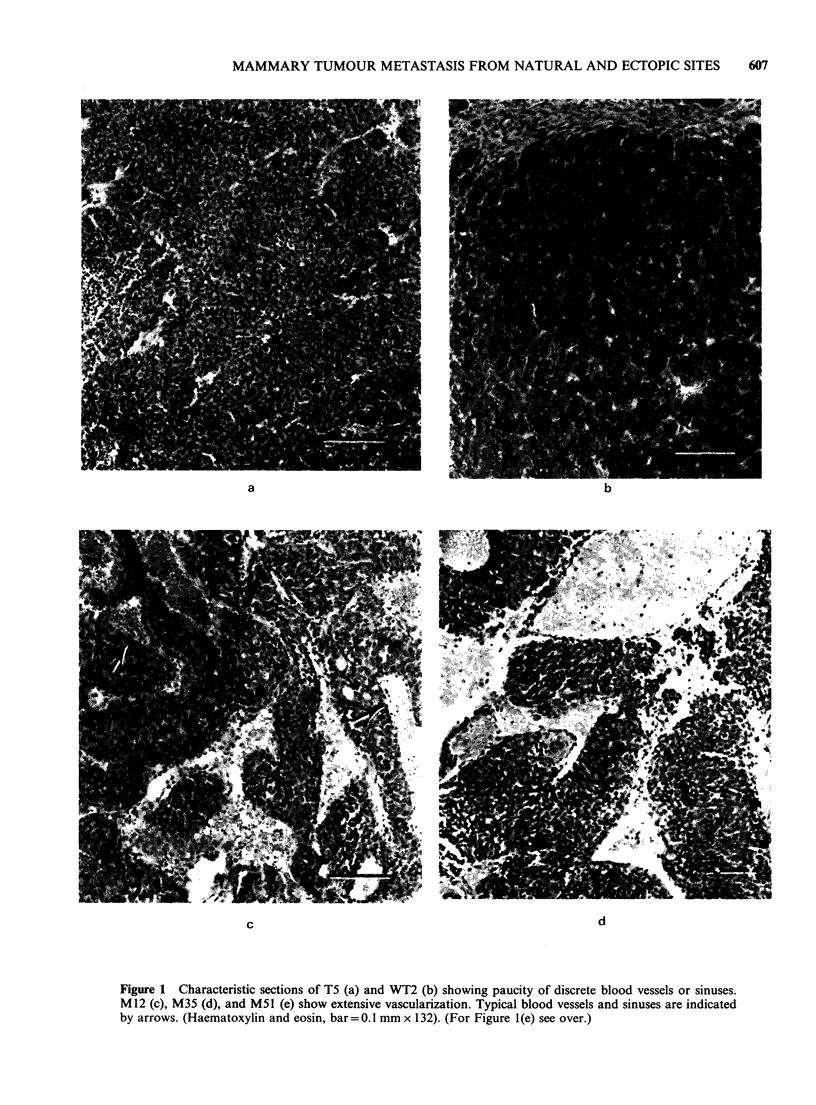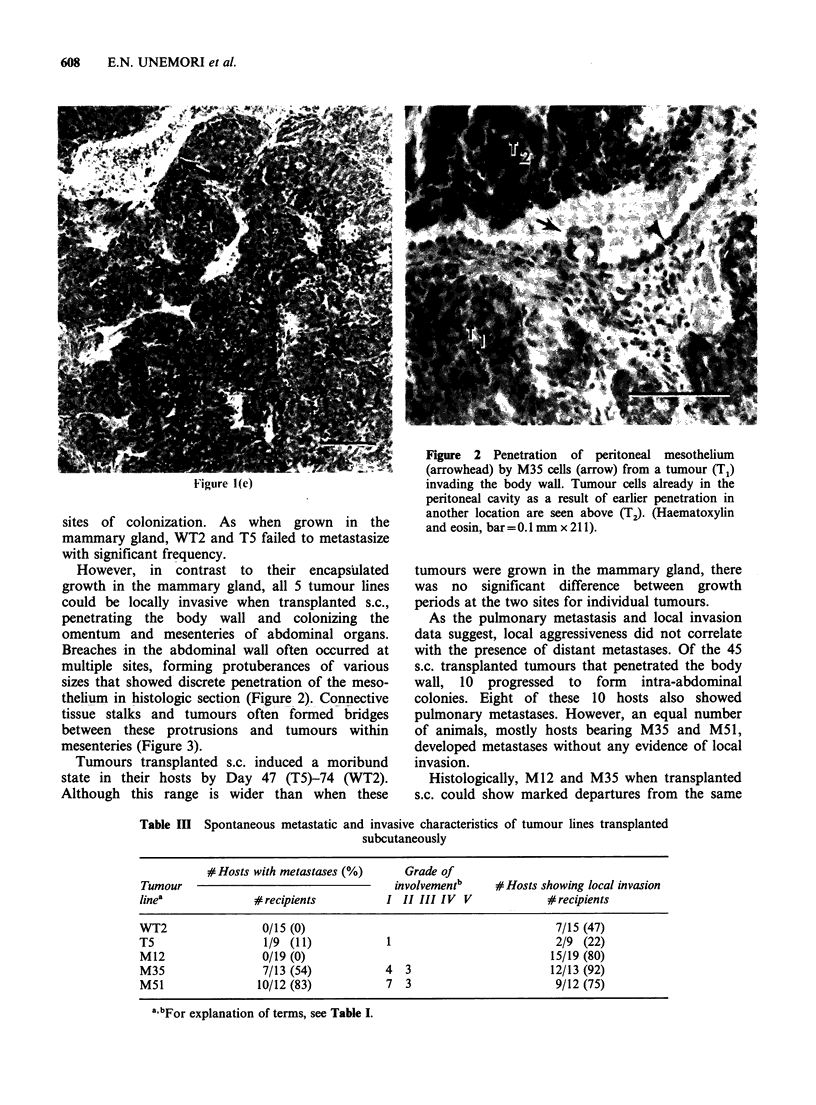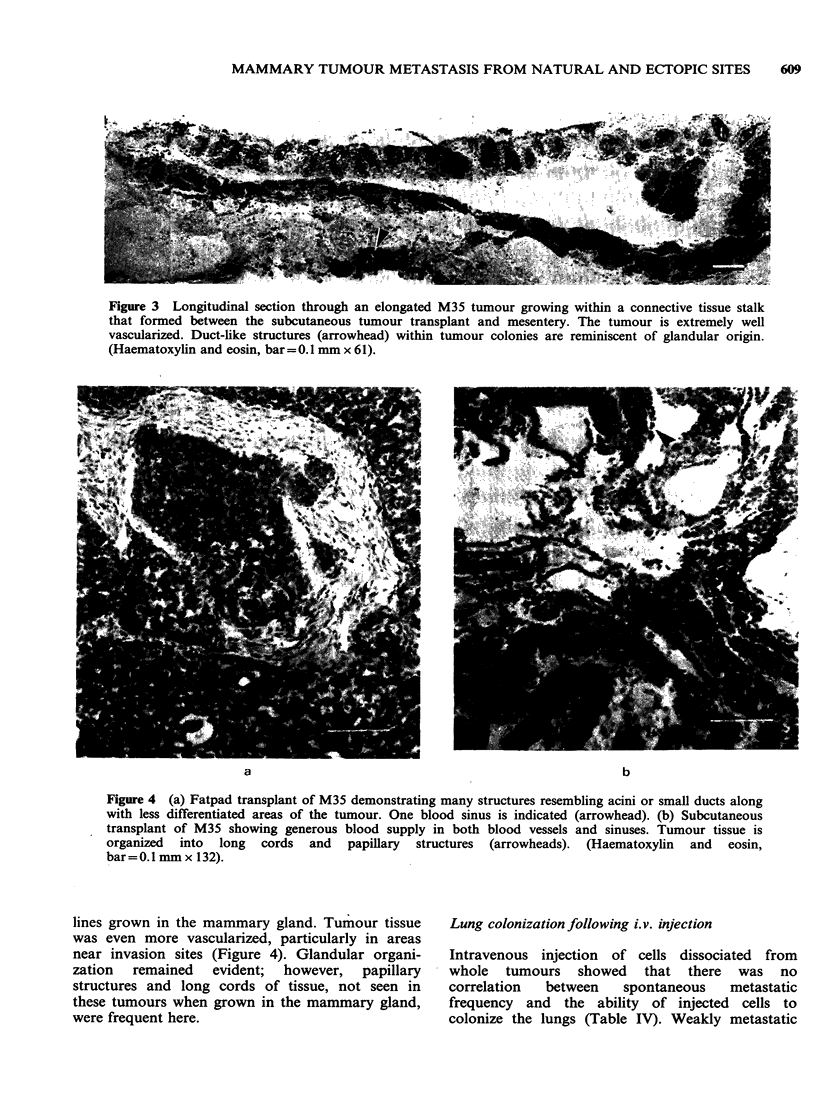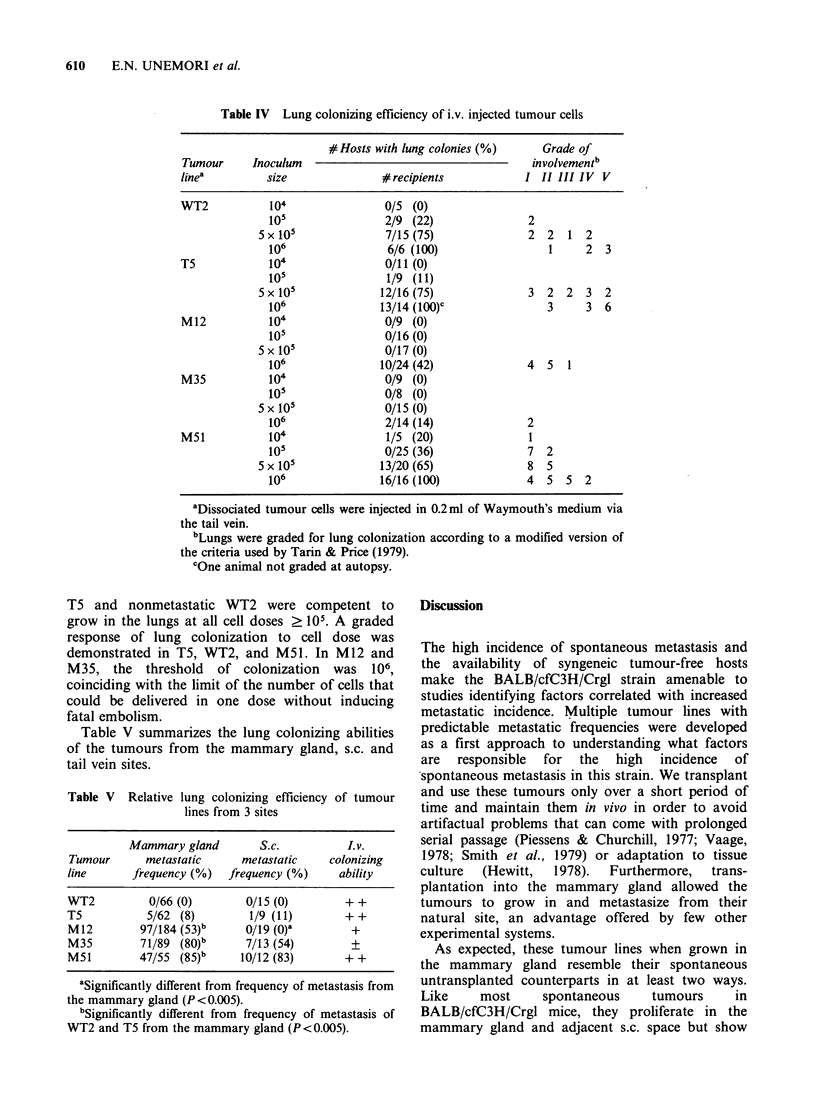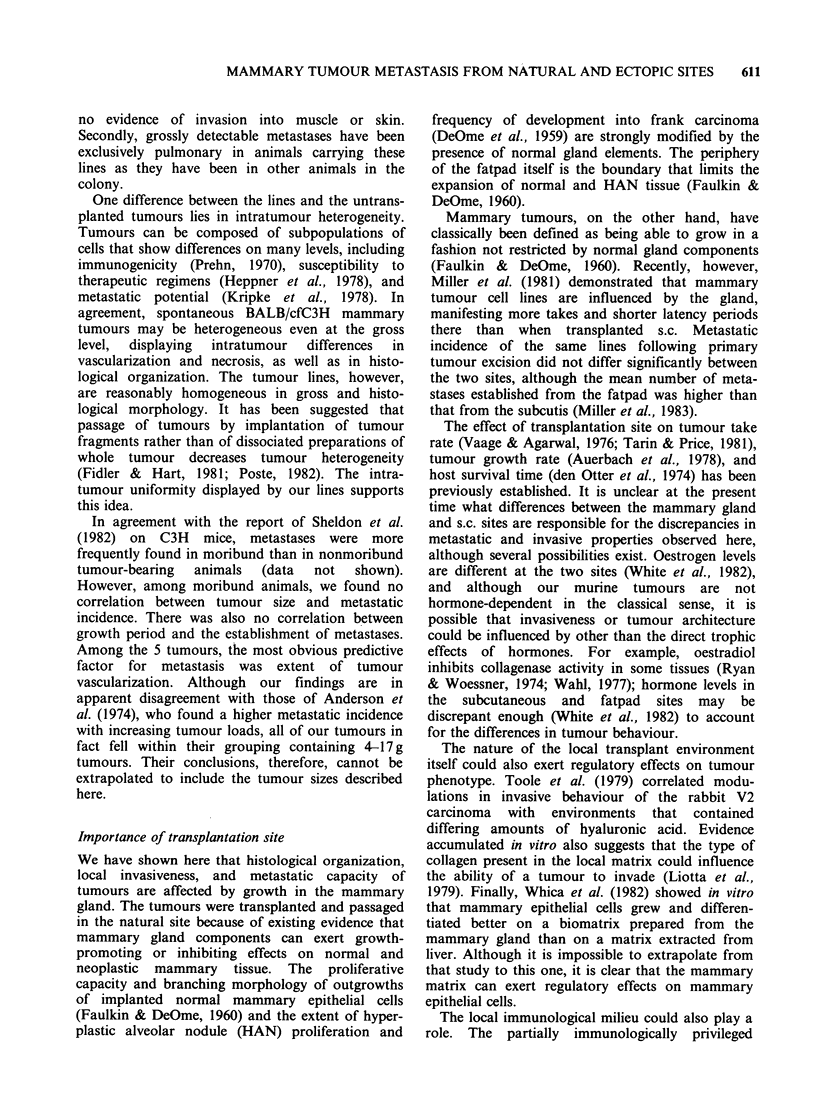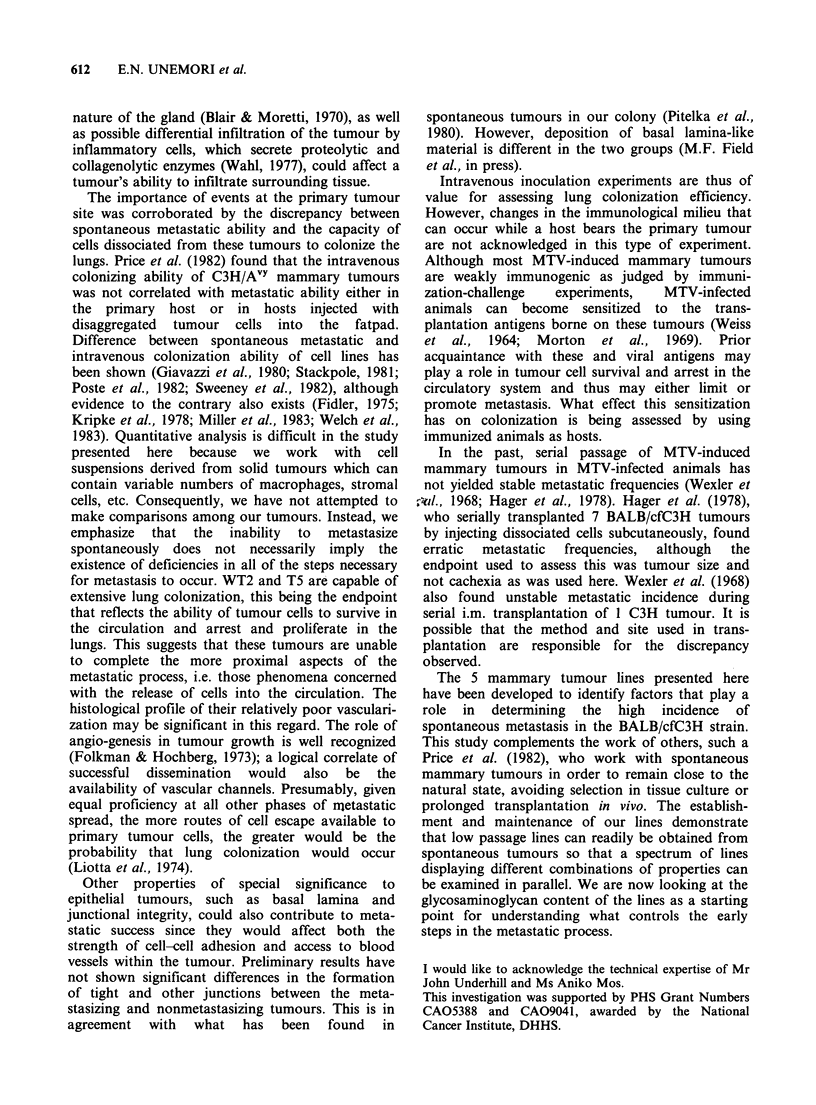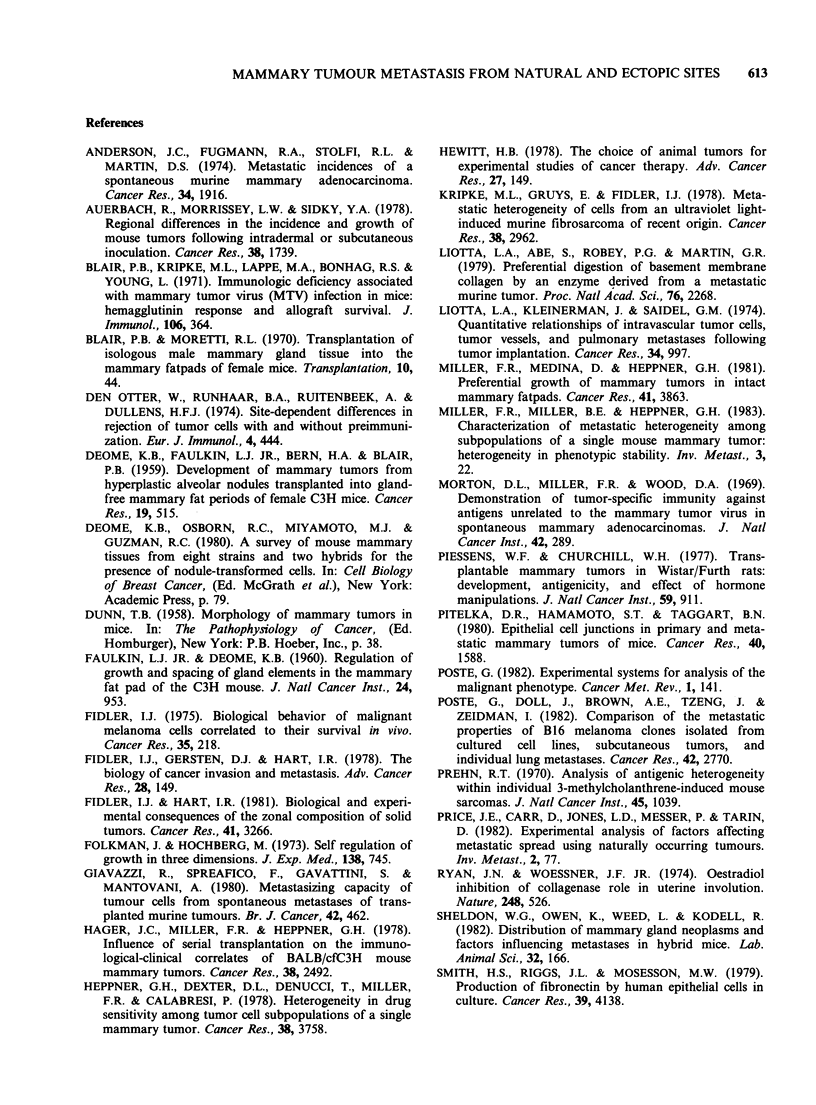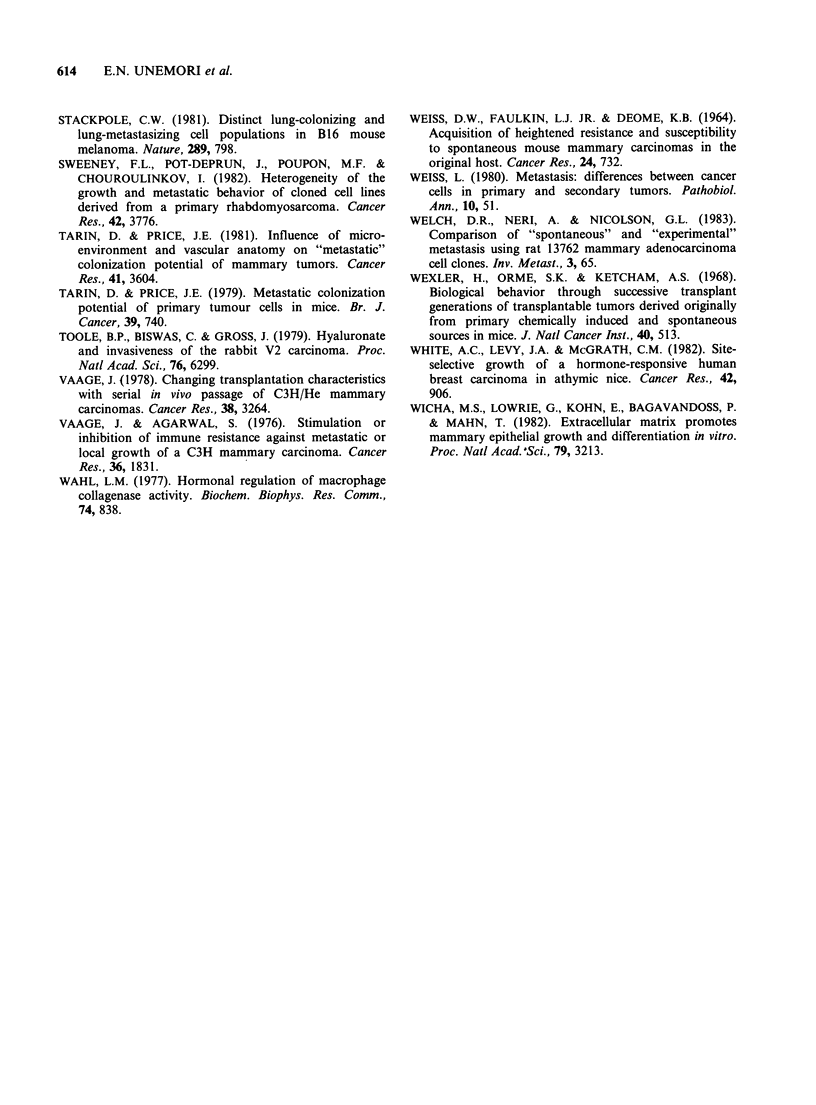# Metastasis of murine mammary tumour lines from the mammary gland and ectopic sites.

**DOI:** 10.1038/bjc.1984.95

**Published:** 1984-05

**Authors:** E. N. Unemori, N. Ways, D. R. Pitelka

## Abstract

**Images:**


*Br. J. Cancer* (1984), **49**, 603–614

### Metastasis of murine mammary tumour lines from the mammary gland and ectopic sites

E.N. Unemori, N. Ways* & D.R. Pitelka

*Department of Zoology and Cancer Research Laboratory, University of California, Berkeley, California 94720, USA.*

**Summary** A murine model of spontaneous metastasis of mammary adenocarcinomas in mice was developed by serial transplantation of spontaneous BALB/cfC3H/Crgl tumours into the mammary gland. Through 8 transplant generations, 5 lines demonstrated maintenance of metastatic phenotype and consistent gross and histological morphology and growth properties. Tumour lines M12, M35, and M51 metastasized from the mammary gland with overall frequencies of 53, 80, and 85%, respectively. Line T5 was weakly metastatic, capable of a minor degree of lung colonization in 8% of hosts, while line WT2 failed to establish any grossly or histologically detectable pulmonary foci.

The significance of the mammary gland as transplant site was shown by comparing the growth and metastasis of these lines in mammary gland with that observed upon subcutaneous transplantation. Subcutaneous metastatic frequency of one tumour line was significantly reduced from that obtained when grown in the mammary gland while histological organization differed markedly in 2 of the tumours. Furthermore, while tumours implanted into the gland grew as well encapsulated masses, the same tumours grown subcutaneously frequently invaded the body wall and occasionally colonized adjacent peritoneal organs and, more often, mesenteries.

Intravenous injection of dissociated tumours further emphasized the importance of events that occur at the primary site. There was no correlation between spontaneous metastatic ability and the capacity to colonize the lung following i.v. inoculation.

This study demonstrates the importance of transplant site in the assessment of metastasis in experimental systems.

Despite the large number of experiments reported in a burgeoning literature on metastasis, only a few tumour systems are actually represented (rev. by Fidler *et al.*, 1978; Weiss, 1980). Although these models have made valuable contributions to understanding the metastatic process, additional ones will be required to represent faithfully all aspects of this phenomenon. It is likely that tumours arising in different organs display somewhat different steps during the formation of metastases, owing to peculiarities in blood flow, architecture, and mechanical stresses of the tissue origin, as well as those of the tumour itself. As an approach to understanding some of the factors that control metastasis of mammary carcinomas, we have developed and characterized 5 mouse mammary tumour lines that vary greatly in their ability to metastasize from their natural site, the mammary gland.

Young virgin BALB/cfC3H/Crgl female mice have virtually no tumours, but old virgin and parous females have mammary tumour incidences of 18% and 93%, respectively (DeOme *et al.*, 1980). Spontaneous pulmonary metastasis detectable by gross and histological inspection occurs in approximately 65% of these tumour-bearers. This murine strain is therefore a generous source of tumour tissue, as well as of young, immunologically competent (Blair *et al.*, 1971) tumour-free hosts, and it was used to initiate and test the mammary tumour lines described here.

The mammary fatpad is known to provide mammary epithelial cells with certain growth-modulating influences not represented at other sites (DeOme *et al.*, 1959; Faulkin & DeOme, 1960; Miller *et al.*, 1981). DeOme *et al.* (1959) showed that normal and preneoplastic mammary cells, which flourish when transplanted into the mammary fatpad, will not grow as well subcutaneously. More recently, Miller *et al.* (1981) demonstrated that mammary tumour cell lines show a preference for the gland, as well. The ease of transplantation into the mammary gland provided the opportunity to allow gland-associated influences to exert their effect on our mammary tumour lines during their development. Furthermore, metastasis was allowed to occur without experimental manipulation and was assessed when the host was moribund. This model therefore assesses

*Present address: Department of Neurobiology, Stanford University School of Medicine, Stanford, California, USA, 94305.
Correspondence: R. Pitelka
Received 15 August 1983; accepted 26 January 1984.

© The Macmillan Press Ltd., 1984

spontaneous metastasis from the natural tumour site.

The prediction that gland-associated factors can contribute to tumour growth and metastasis was tested and affirmed by s.c. transplantation and i.v. inoculation of these tumours.

### Materials and methods

### *Animals*

Female BALB/cfC3H/Crgl (Mammary Tumour Virus (MTV)+) mice were obtained from the inbred mouse colony maintained at the Cancer Research Laboratory, Berkeley, California. Food pellets (Wayne Lab/Blox F-G, Allied Mills, Inc., Chicago, Illinois) and water were available *ad libitum*. Multiparous and virgin animals that spontaneously developed single mammary tumours were sources of tumour tissue. Mice carrying primary or transplanted tumours as part of this study were killed by neck fracture. At autopsy, tumours were measured, grossly characterized, and sampled for histology. Organs were inspected for the presence of metastatic lesions.

### *Assessment of metastasis*

Lungs and peritoneal organs were examined for metastatic involvement. Organs were sliced in several planes and examined either grossly or under the dissecting microscope for the presence and extent of tumour invasion.

Degree of replacement of lung tissue by tumour was graded according to the system of Tarin & Price (1979), modified as follows.

| | |
|---|---|
| Grade I | Few small deposits ($\leq 10$, $\leq 1\,mm^3$) |
| Grade II | More extensive deposition than Grade I but less than 1/4 of lung tissue replaced |
| Grade III | 1/4–1/2 of lung tissue replaced |
| Grade IV | 1/2–3/4 of lung tissue replaced |
| Grade V | More than 3/4 of lung tissue replaced |

In lobes that showed definite tumour invasion, histological analysis was not routinely done, so that any error in grading tends to underestimate rather than overestimate lung replacement. In cases where tumour deposition was suspected, histological examination was performed; if foci were discovered, these lungs were recorded as Grade I.

### *Tumour passage*

Tumour-bearing BALB/cfC3H/Crgl mice have a spontaneous metastatic incidence of about 65%. In our experience, metastases have been exclusively pulmonary. Tumours obtained from these mice were divided into 2 groups. Group I consisted of tumours that produced no detectable metastases in the primary host. Group II contained tumours that had established fairly extensive metastases (Grades II–V). Tumours that were weakly metastatic and those in animals with multiple mammary tumours were not used in this study. Tumours were preferentially taken from moribund animals to increase the likelihood that metastatic potential be expressed in the primary host (Anderson *et al.*, 1974; Sheldon *et al.*, 1982).

Tumours in Group I were used to initiate non-metastasizing tumour lines in the following manner. Pieces ($1\,mm^3$) from a small area of the tumour were rinsed briefly in saline, blotted, then transplanted into the right no. 4 mammary glands of several normal virgin BALB/cfC3H/Crgl female mice. These virgins were used when 2–3 months old since it is known that they rarely develop their own tumours before 9 months of age (DeOme *et al.*, 1980). After tumour implantation, animals were observed for 1–3 months and sacrificed when they became moribund. At autopsy, metastatic involvement was recorded. The tumour was measured, minced, and $1\,mm^3$ pieces were transplanted into the mammary glands of 2–3 month-old virgin recipients. Metastatic frequency, histology, and gross morphology of the tumours were assessed at each generation.

Tumours classified in Group II were used to establish metastasizing tumour lines. Pieces of the primary tumour $1\,mm^3$ in size were transplanted. Hosts were sacrificed when moribund and checked for tumour spread. Tumours were serially transplanted as $1\,mm^3$ pieces and metastatic frequency, histology, and gross morphology recorded at each generation.

### *Subcutaneous transplantation*

Transplanted tumours were harvested from the mammary gland and $1\,mm^3$ pieces were implanted into the dorsal s.c. space at the level of the no. 4 fatpad in 2–3-month-old BALB/cfC3H/Crgl virgin mice. To avoid abrasion of the muscle of skin during the implantation, the skin was first separated from the underlying muscle with a cotton swab. Mice were sacrificed when moribund and checked for metastatic involvement. Tumours were measured and sampled for histology.

### *Intravenous injection*

Tumour lines developed by the method described above were used for i.v. injection experiments. Tumours grown in the mammary gland were excised, weighed, and minced with a sterile razor blade.

Tumours were rinsed briefly in saline before dissociation. Dissociating medium consisted of 0.1% collagenase (Sigma, Type I, 210 U $mg^{-1}$, St. Louis, Missouri) and 0.4% bovine serum albumin (Reheis, Fraction V, Phoenix, Arizona) prepared in Waymouth's medium (MB 752/1, GIBCO, Grand Island, New York). Ten ml of dissociating medium was added for each g of minced tumour tissue and dissociation accomplished at 37°C while shaking. At the end of 1 1/2 h, DNase (prepared in Waymouth's medium) was added to a final concentration of 0.009% and the mixture incubated for 10–15 min at room temperature. The suspension was poured through sterile Dacron (approximate mesh size 0.3 $mm^2$) for removal of residual large pieces of tissue. The filtrate was centrifuged at 130 *g* for 8–10 min and the pellet washed once in Waymouth's medium. Cells and small clumps that passed consecutively through 150 $\mu m$ and 10 $\mu m$ Nitex filters were counted and their viability assessed using trypan blue. Cell preparations showing <90% viability were discarded.

Cells were adjusted to $5 \times 10^4$ to $5 \times 10^6$ cells $ml^{-1}$ in Waymouth's medium. Cell suspensions were occasionally stored overnight at 4°C, with no effect on colonization. Viability and cell number were assessed immediately prior to use. Sterile technique was used throughout the cell dissociation and dilution procedures.

The cell preparation (0.2 ml) was injected into the tail veins of 2- to 3-month-old BALB/cfC3H/Crgl virgin female mice. Recipients were sacrificed in 8 weeks or earlier if death appeared imminent. At autopsy, lungs and abdominal organs were checked for tumours; occasionally brains were also examined. Degree of lung involvement was graded according to the system described above.

*Histology*

Pieces of tumour tissue were reserved for histology throughout serial transplantation. Pieces (3–5 $mm^3$) were fixed overnight in Bouin's fixative. Sections (7 $\mu m$) were stained with haematoxylin and eosin. Tumours were classified according to the criteria of Dunn (1958).

*Statistical analysis*

The data were analyzed for significance using the chi-square test with Yates' correction for continuity.

### Results

Five tumour lines initiated using this transplantation method will be described. These consist of one nonmetastasizing line, one line with low metastatic frequency, and 3 lines with high metastatic frequency that have been passaged in the mammary gland and characterized through 8 generations.

*Growth in and metastasis from the mammary gland*

Nonmetastasizing and weakly metastatic tumour lines WT2 and T5 originated from Group I tumours found in 2 multiparous females. In 8 generations, 66 WT2 hosts have remained metastasis-free (Table I). The overall metastatic capacity of line T5 through 8 generations has been 8% (5/62) (Table I). In 4 of the 5 hosts displaying metastasis, 1 pulmonary nodule measuring <1 $mm^3$ was the extent of colonization. Metastatic involvement in all 5 affected mice was minor, as indicated by the grade of I in all cases.

**Table I** Transplant characteristics of tumour lines WT2 and T5

| *Transplant Generation*[a] | *# Mice with metastasis(%)* *# WT2 recipients* | *# Mice with metastasis (%)* *# T5 recipients* | *Grade of Involvement*[b] *I* | *II* | *III* | *IV* | *V* |
|---|---|---|---|---|---|---|---|
| 1 | 0/3 (0) | 2/4 (50) | 2 | | | | |
| 2 | 0/10 (0) | 0/4 (0) | | | | | |
| 3 | 0/3 (0) | 0/18 (0) | | | | | |
| 4 | 0/5 (0) | 2/17 (12) | 2 | | | | |
| 5 | 0/11 (0) | 0/7 (0) | | | | | |
| 6 | 0/11 (0) | 0/6 (0) | | | | | |
| 7 | 0/8 (0) | 1/6 (17) | 1 | | | | |
| 8 | 0/15 (0) | 0/8 (0) | | | | | |
| Total | 0/66 (0) | 5/62 (8) | 5 | | | | |

[a]Tumour pieces 1 $mm^3$ in size were transplanted into the mammary glands of 2- to 3-month old BALB/cfC3H recipients. When moribund, animals were sacrificed and inspected by gross and histological examination for metastasis.

[b]Lungs were graded for metastatic involvement according to a modified version of the criteria used by Tarin & Price (1979).

**Table II** Transplant characteristics of tumour lines M12, M35, and M51

| Transplant generation[a] | # Mice w/metastases (%) # M12 recipients | Grade of involvement[b] I | II | III | IV | V | # Mice w/metastases (%) # M35 recipients | Grade of involvement[b] I | II | III | IV | V | # Mice w/metastases (%) # M51 recipients | Grade of involvement[b] I | II | III | IV | V |
|---|---|---|---|---|---|---|---|---|---|---|---|---|---|---|---|---|---|---|
| 1 | 4/6 (66) | 1 | 1 | | | 2 | 8/11 (72) | 3 | 2 | 1 | 2 | | 3/5 (60) | 1 | 1 | 1 | | |
| 2 | 10/18 (56) | 3 | 4 | 1 | 2 | | 12/15 (80) | 3 | 5 | 4 | | | 3/5 (60) | 1 | 1 | 1 | | |
| 3 | 23/58 (40) | 9 | 4 | 6 | 2 | 2 | 15/23 (65) | 3 | 6 | 4 | 2 | | 5/5 (100) | | | 2 | 2 | 1 |
| 4 | 29/60 (48) | 15 | 5 | 7 | 2 | | 11/11 (100) | 5 | 4 | 2 | | | 6/6 (100) | 1 | | 4 | 1 | |
| 5 | 10/17 (59) | 2 | 4 | 3 | 1 | | 6/9 (67) | 1 | 2 | 2 | 1 | | 10/12 (83) | 1 | 2 | 4 | 2 | 1 |
| 6 | 5/5 (100) | | 1 | 2 | 2 | | 7/8 (88) | 1 | 2 | 4 | | | 7/8 (75) | 3 | 2 | 2 | | |
| 7 | 12/12 (100) | | 4 | 6 | 2 | | 8/8 (100) | 4 | 2 | 2 | | | 4/4 (100) | | 3 | 1 | | |
| 8 | 4/8 (50) | 1 | 1 | 1 | 1 | | 6/6 (100) | 4 | 1 | 1 | | | 9/10 (90) | 3 | 3 | 3 | | |
| Total | 97/184 (53) | 31 | 24 | 26 | 12 | 4 | 71/89 (80) | 24 | 24 | 20 | 5 | 0 | 47/55 (85) | 10 | 12 | 18 | 5 | 2 |

[a,b]For explanation of terms, see **Table I**.

Line M12 was initiated from a pulmonary metastasis of a Group II tumour that arose in an old virgin female. The metastatic incidence of this line was 53% (97/184) over 8 generations (Table II). A primary tumour from a multiparous female whose normal lung tissue was almost obliterated by metastatic growth was used to initiate line M35. Upon serial transplantation, it established lung tumours in 71/89 (80%) recipients over 8 generations (Table II). The average degree of lung replacement in affected animals was slightly less extensive than that in animals carrying M12, but many more animals were thus affected. The tumour that gave rise to line M51 arose in an old virgin mouse. Extensive metastatic involvement of all pulmonary lobes was observed. In 8 generations, 47/55 (85%) hosts showed secondary involvement. Although M35 and M51 have similar metastatic frequencies, M51 gave rise to more extensive pulmonary colonization with more than half of affected animals showing lung involvement of grades III–V.

All 5 tumour lines caused a moribund state in their hosts by day 47 (T5)–60 (M51). There was no correlation between metastasizing ability and period of growth in hosts.

Tumour dimensions along the longest and shortest axes in the frontal plane were measured in moribund mice. Average tumour sizes ranged from 30 × 24 mm (M51) to 42 × 31 mm (T5). There was no correlation between metastasizing ability and tumour size at autopsy or between growth period and tumour size. Except in cases where lung involvement was graded V, cachexia induced by the primary tumour load was the cause of death.

In addition to stability in metastatic capacity, gross morphology as exemplified by degree and type of necrosis, extent of vascularization, and tissue integrity has been consistent from generation to generation in all 5 tumours. Histologically, all lines are Type B carcinomas with varying degrees of glandular organization into acini or cords, stromal contribution, lymphocytic infiltration, and presence of blood vessels and sinuses. The metastasizing tumours are significantly more vascularized than their nonmetastasizing counterparts (Figure 1). The histological picture of each tumour has remained stable with serial transplantation. All tumours grew as well encapsulated masses; invasion into overlying skin, underlying muscle or bone, or adjacent body wall was not apparent.

*Growth in and metastasis from a s.c. site*

Subcutaneous transplantation of these tumours resulted in a significant reduction in metastatic ability of one line, M12 ($P < 0.005$) (Table III). M35 and M51 remained metastatic in a majority of their hosts and the lungs remained the exclusive

**Figure 1** Characteristic sections of T5 (a) and WT2 (b) showing paucity of discrete blood vessels or sinuses. M12 (c), M35 (d), and M51 (e) show extensive vascularization. Typical blood vessels and sinuses are indicated by arrows. (Haematoxylin and eosin, bar = 0.1 mm × 132). (For Figure 1(e) see over.)

Figure 1(e)

**Figure 2** Penetration of peritoneal mesothelium (arrowhead) by M35 cells (arrow) from a tumour ($T_1$) invading the body wall. Tumour cells already in the peritoneal cavity as a result of earlier penetration in another location are seen above ($T_2$). (Haematoxylin and eosin, bar = 0.1 mm × 211).

sites of colonization. As when grown in the mammary gland, WT2 and T5 failed to metastasize with significant frequency.

However, in contrast to their encapsulated growth in the mammary gland, all 5 tumour lines could be locally invasive when transplanted s.c., penetrating the body wall and colonizing the omentum and mesenteries of abdominal organs. Breaches in the abdominal wall often occurred at multiple sites, forming protuberances of various sizes that showed discrete penetration of the mesothelium in histologic section (Figure 2). Connective tissue stalks and tumours often formed bridges between these protrusions and tumours within mesenteries (Figure 3).

Tumours transplanted s.c. induced a moribund state in their hosts by Day 47 (T5)–74 (WT2). Although this range is wider than when these tumours were grown in the mammary gland, there was no significant difference between growth periods at the two sites for individual tumours.

As the pulmonary metastasis and local invasion data suggest, local aggressiveness did not correlate with the presence of distant metastases. Of the 45 s.c. transplanted tumours that penetrated the body wall, 10 progressed to form intra-abdominal colonies. Eight of these 10 hosts also showed pulmonary metastases. However, an equal number of animals, mostly hosts bearing M35 and M51, developed metastases without any evidence of local invasion.

Histologically, M12 and M35 when transplanted s.c. could show marked departures from the same

**Table III** Spontaneous metastatic and invasive characteristics of tumour lines transplanted subcutaneously

| Tumour line[a] | *# Hosts with metastases (%)* / *# recipients* | *Grade of involvement*[b] *I* | *II* | *III* | *IV* | *V* | *# Hosts showing local invasion* / *# recipients* |
|---|---|---|---|---|---|---|---|
| WT2 | 0/15 (0) | | | | | | 7/15 (47) |
| T5 | 1/9 (11) | 1 | | | | | 2/9 (22) |
| M12 | 0/19 (0) | | | | | | 15/19 (80) |
| M35 | 7/13 (54) | 4 | 3 | | | | 12/13 (92) |
| M51 | 10/12 (83) | 7 | 3 | | | | 9/12 (75) |

[a,b]For explanation of terms, see **Table I**.

**Figure 3** Longitudinal section through an elongated M35 tumour growing within a connective tissue stalk that formed between the subcutaneous tumour transplant and mesentery. The tumour is extremely well vascularized. Duct-like structures (arrowhead) within tumour colonies are reminiscent of glandular origin. (Haematoxylin and eosin, bar = 0.1 mm × 61).

a b

**Figure 4** (a) Fatpad transplant of M35 demonstrating many structures resembling acini or small ducts along with less differentiated areas of the tumour. One blood sinus is indicated (arrowhead). (b) Subcutaneous transplant of M35 showing generous blood supply in both blood vessels and sinuses. Tumour tissue is organized into long cords and papillary structures (arrowheads). (Haematoxylin and eosin, bar = 0.1 mm × 132).

lines grown in the mammary gland. Tumour tissue was even more vascularized, particularly in areas near invasion sites (Figure 4). Glandular organization remained evident; however, papillary structures and long cords of tissue, not seen in these tumours when grown in the mammary gland, were frequent here.

*Lung colonization following i.v. injection*

Intravenous injection of cells dissociated from whole tumours showed that there was no correlation between spontaneous metastatic frequency and the ability of injected cells to colonize the lungs (Table IV). Weakly metastatic

**Table IV** Lung colonizing efficiency of i.v. injected tumour cells

| *Tumour line*[a] | *Inoculum size* | *# Hosts with lung colonies (%)* / *# recipients* | *Grade of involvement*[b] I | II | III | IV | V |
|---|---|---|---|---|---|---|---|
| WT2 | $10^4$ | 0/5 (0) | | | | | |
| | $10^5$ | 2/9 (22) | 2 | | | | |
| | $5 \times 10^5$ | 7/15 (75) | 2 | 2 | 1 | 2 | |
| | $10^6$ | 6/6 (100) | | 1 | | 2 | 3 |
| T5 | $10^4$ | 0/11 (0) | | | | | |
| | $10^5$ | 1/9 (11) | | | | | |
| | $5 \times 10^5$ | 12/16 (75) | 3 | 2 | 2 | 3 | 2 |
| | $10^6$ | 13/14 (100)[c] | | 3 | | 3 | 6 |
| M12 | $10^4$ | 0/9 (0) | | | | | |
| | $10^5$ | 0/16 (0) | | | | | |
| | $5 \times 10^5$ | 0/17 (0) | | | | | |
| | $10^6$ | 10/24 (42) | 4 | 5 | 1 | | |
| M35 | $10^4$ | 0/9 (0) | | | | | |
| | $10^5$ | 0/8 (0) | | | | | |
| | $5 \times 10^5$ | 0/15 (0) | | | | | |
| | $10^6$ | 2/14 (14) | 2 | | | | |
| M51 | $10^4$ | 1/5 (20) | 1 | | | | |
| | $10^5$ | 0/25 (36) | 7 | 2 | | | |
| | $5 \times 10^5$ | 13/20 (65) | 8 | 5 | | | |
| | $10^6$ | 16/16 (100) | 4 | 5 | 5 | 2 | |

[a]Dissociated tumour cells were injected in 0.2 ml of Waymouth's medium via the tail vein.

[b]Lungs were graded for lung colonization according to a modified version of the criteria used by Tarin & Price (1979).

[c]One animal not graded at autopsy.

T5 and nonmetastatic WT2 were competent to grow in the lungs at all cell doses $\geq 10^5$. A graded response of lung colonization to cell dose was demonstrated in T5, WT2, and M51. In M12 and M35, the threshold of colonization was $10^6$, coinciding with the limit of the number of cells that could be delivered in one dose without inducing fatal embolism.

Table V summarizes the lung colonizing abilities of the tumours from the mammary gland, s.c. and tail vein sites.

**Table V** Relative lung colonizing efficiency of tumour lines from 3 sites

| *Tumour line* | *Mammary gland metastatic frequency* (%) | *S.c. metastatic frequency* (%) | *I.v. colonizing ability* |
|---|---|---|---|
| WT2 | 0/66 (0) | 0/15 (0) | + + |
| T5 | 5/62 (8) | 1/9 (11) | + + |
| M12 | 97/184 (53)[b] | 0/19 (0)[a] | + |
| M35 | 71/89 (80)[b] | 7/13 (54) | ± |
| M51 | 47/55 (85)[b] | 10/12 (83) | + + |

[a]Significantly different from frequency of metastasis from the mammary gland ($P < 0.005$).

[b]Significantly different from frequency of metastasis of WT2 and T5 from the mammary gland ($P < 0.005$).

### Discussion

The high incidence of spontaneous metastasis and the availability of syngeneic tumour-free hosts make the BALB/cfC3H/Crgl strain amenable to studies identifying factors correlated with increased metastatic incidence. Multiple tumour lines with predictable metastatic frequencies were developed as a first approach to understanding what factors are responsible for the high incidence of spontaneous metastasis in this strain. We transplant and use these tumours only over a short period of time and maintain them *in vivo* in order to avoid artifactual problems that can come with prolonged serial passage (Piessens & Churchill, 1977; Vaage, 1978; Smith *et al.*, 1979) or adaptation to tissue culture (Hewitt, 1978). Furthermore, transplantation into the mammary gland allowed the tumours to grow in and metastasize from their natural site, an advantage offered by few other experimental systems.

As expected, these tumour lines when grown in the mammary gland resemble their spontaneous untransplanted counterparts in at least two ways. Like most spontaneous tumours in BALB/cfC3H/Crgl mice, they proliferate in the mammary gland and adjacent s.c. space but show no evidence of invasion into muscle or skin. Secondly, grossly detectable metastases have been exclusively pulmonary in animals carrying these lines as they have been in other animals in the colony.

One difference between the lines and the untransplanted tumours lies in intratumour heterogeneity. Tumours can be composed of subpopulations of cells that show differences on many levels, including immunogenicity (Prehn, 1970), susceptibility to therapeutic regimens (Heppner *et al.*, 1978), and metastatic potential (Kripke *et al.*, 1978). In agreement, spontaneous BALB/cfC3H mammary tumours may be heterogeneous even at the gross level, displaying intratumour differences in vascularization and necrosis, as well as in histological organization. The tumour lines, however, are reasonably homogeneous in gross and histological morphology. It has been suggested that passage of tumours by implantation of tumour fragments rather than of dissociated preparations of whole tumour decreases tumour heterogeneity (Fidler & Hart, 1981; Poste, 1982). The intratumour uniformity displayed by our lines supports this idea.

In agreement with the report of Sheldon *et al.* (1982) on C3H mice, metastases were more frequently found in moribund than in nonmoribund tumour-bearing animals (data not shown). However, among moribund animals, we found no correlation between tumour size and metastatic incidence. There was also no correlation between growth period and the establishment of metastases. Among the 5 tumours, the most obvious predictive factor for metastasis was extent of tumour vascularization. Although our findings are in apparent disagreement with those of Anderson *et al.* (1974), who found a higher metastatic incidence with increasing tumour loads, all of our tumours in fact fell within their grouping containing 4–17 g tumours. Their conclusions, therefore, cannot be extrapolated to include the tumour sizes described here.

*Importance of transplantation site*

We have shown here that histological organization, local invasiveness, and metastatic capacity of tumours are affected by growth in the mammary gland. The tumours were transplanted and passaged in the natural site because of existing evidence that mammary gland components can exert growth-promoting or inhibiting effects on normal and neoplastic mammary tissue. The proliferative capacity and branching morphology of outgrowths of implanted normal mammary epithelial cells (Faulkin & DeOme, 1960) and the extent of hyperplastic alveolar nodule (HAN) proliferation and frequency of development into frank carcinoma (DeOme *et al.*, 1959) are strongly modified by the presence of normal gland elements. The periphery of the fatpad itself is the boundary that limits the expansion of normal and HAN tissue (Faulkin & DeOme, 1960).

Mammary tumours, on the other hand, have classically been defined as being able to grow in a fashion not restricted by normal gland components (Faulkin & DeOme, 1960). Recently, however, Miller *et al.* (1981) demonstrated that mammary tumour cell lines are influenced by the gland, manifesting more takes and shorter latency periods there than when transplanted s.c. Metastatic incidence of the same lines following primary tumour excision did not differ significantly between the two sites, although the mean number of metastases established from the fatpad was higher than that from the subcutis (Miller *et al.*, 1983).

The effect of transplantation site on tumour take rate (Vaage & Agarwal, 1976; Tarin & Price, 1981), tumour growth rate (Auerbach *et al.*, 1978), and host survival time (den Otter *et al.*, 1974) has been previously established. It is unclear at the present time what differences between the mammary gland and s.c. sites are responsible for the discrepancies in metastatic and invasive properties observed here, although several possibilities exist. Oestrogen levels are different at the two sites (White *et al.*, 1982), and although our murine tumours are not hormone-dependent in the classical sense, it is possible that invasiveness or tumour architecture could be influenced by other than the direct trophic effects of hormones. For example, oestradiol inhibits collagenase activity in some tissues (Ryan & Woessner, 1974; Wahl, 1977); hormone levels in the subcutaneous and fatpad sites may be discrepant enough (White *et al.*, 1982) to account for the differences in tumour behaviour.

The nature of the local transplant environment itself could also exert regulatory effects on tumour phenotype. Toole *et al.* (1979) correlated modulations in invasive behaviour of the rabbit V2 carcinoma with environments that contained differing amounts of hyaluronic acid. Evidence accumulated *in vitro* also suggests that the type of collagen present in the local matrix could influence the ability of a tumour to invade (Liotta *et al.*, 1979). Finally, Whica *et al.* (1982) showed *in vitro* that mammary epithelial cells grew and differentiated better on a biomatrix prepared from the mammary gland than on a matrix extracted from liver. Although it is impossible to extrapolate from that study to this one, it is clear that the mammary matrix can exert regulatory effects on mammary epithelial cells.

The local immunological milieu could also play a role. The partially immunologically privileged nature of the gland (Blair & Moretti, 1970), as well as possible differential infiltration of the tumour by inflammatory cells, which secrete proteolytic and collagenolytic enzymes (Wahl, 1977), could affect a tumour's ability to infiltrate surrounding tissue.

The importance of events at the primary tumour site was corroborated by the discrepancy between spontaneous metastatic ability and the capacity of cells dissociated from these tumours to colonize the lungs. Price *et al.* (1982) found that the intravenous colonizing ability of C3H/$A^{vy}$ mammary tumours was not correlated with metastatic ability either in the primary host or in hosts injected with disaggregated tumour cells into the fatpad. Difference between spontaneous metastatic and intravenous colonization ability of cell lines has been shown (Giavazzi *et al.*, 1980; Stackpole, 1981; Poste *et al.*, 1982; Sweeney *et al.*, 1982), although evidence to the contrary also exists (Fidler, 1975; Kripke *et al.*, 1978; Miller *et al.*, 1983; Welch *et al.*, 1983). Quantitative analysis is difficult in the study presented here because we work with cell suspensions derived from solid tumours which can contain variable numbers of macrophages, stromal cells, etc. Consequently, we have not attempted to make comparisons among our tumours. Instead, we emphasize that the inability to metastasize spontaneously does not necessarily imply the existence of deficiencies in all of the steps necessary for metastasis to occur. WT2 and T5 are capable of extensive lung colonization, this being the endpoint that reflects the ability of tumour cells to survive in the circulation and arrest and proliferate in the lungs. This suggests that these tumours are unable to complete the more proximal aspects of the metastatic process, i.e. those phenomena concerned with the release of cells into the circulation. The histological profile of their relatively poor vascularization may be significant in this regard. The role of angio-genesis in tumour growth is well recognized (Folkman & Hochberg, 1973); a logical correlate of successful dissemination would also be the availability of vascular channels. Presumably, given equal proficiency at all other phases of metastatic spread, the more routes of cell escape available to primary tumour cells, the greater would be the probability that lung colonization would occur (Liotta *et al.*, 1974).

Other properties of special significance to epithelial tumours, such as basal lamina and junctional integrity, could also contribute to metastatic success since they would affect both the strength of cell–cell adhesion and access to blood vessels within the tumour. Preliminary results have not shown significant differences in the formation of tight and other junctions between the metastasizing and nonmetastasizing tumours. This is in agreement with what has been found in spontaneous tumours in our colony (Pitelka *et al.*, 1980). However, deposition of basal lamina-like material is different in the two groups (M.F. Field *et al.*, in press).

Intravenous inoculation experiments are thus of value for assessing lung colonization efficiency. However, changes in the immunological milieu that can occur while a host bears the primary tumour are not acknowledged in this type of experiment. Although most MTV-induced mammary tumours are weakly immunogenic as judged by immunization-challenge experiments, MTV-infected animals can become sensitized to the transplantation antigens borne on these tumours (Weiss *et al.*, 1964; Morton *et al.*, 1969). Prior acquaintance with these and viral antigens may play a role in tumour cell survival and arrest in the circulatory system and thus may either limit or promote metastasis. What effect this sensitization has on colonization is being assessed by using immunized animals as hosts.

In the past, serial passage of MTV-induced mammary tumours in MTV-infected animals has not yielded stable metastatic frequencies (Wexler *et al.*, 1968; Hager *et al.*, 1978). Hager *et al.* (1978), who serially transplanted 7 BALB/cfC3H tumours by injecting dissociated cells subcutaneously, found erratic metastatic frequencies, although the endpoint used to assess this was tumour size and not cachexia as was used here. Wexler *et al.* (1968) also found unstable metastatic incidence during serial i.m. transplantation of 1 C3H tumour. It is possible that the method and site used in transplantation are responsible for the discrepancy observed.

The 5 mammary tumour lines presented here have been developed to identify factors that play a role in determining the high incidence of spontaneous metastasis in the BALB/cfC3H strain. This study complements the work of others, such a Price *et al.* (1982), who work with spontaneous mammary tumours in order to remain close to the natural state, avoiding selection in tissue culture or prolonged transplantation *in vivo*. The establishment and maintenance of our lines demonstrate that low passage lines can readily be obtained from spontaneous tumours so that a spectrum of lines displaying different combinations of properties can be examined in parallel. We are now looking at the glycosaminoglycan content of the lines as a starting point for understanding what controls the early steps in the metastatic process.

I would like to acknowledge the technical expertise of Mr John Underhill and Ms Aniko Mos.
This investigation was supported by PHS Grant Numbers CAO5388 and CAO9041, awarded by the National Cancer Institute, DHHS.

### References

ANDERSON, J.C., FUGMANN, R.A., STOLFI, R.L. & MARTIN, D.S. (1974). Metastatic incidences of a spontaneous murine mammary adenocarcinoma. *Cancer Res.*, **34**, 1916.

AUERBACH, R., MORRISSEY, L.W. & SIDKY, Y.A. (1978). Regional differences in the incidence and growth of mouse tumors following intradermal or subcutaneous inoculation. *Cancer Res.*, **38**, 1739.

BLAIR, P.B., KRIPKE, M.L., LAPPE, M.A., BONHAG, R.S. & YOUNG, L. (1971). Immunologic deficiency associated with mammary tumor virus (MTV) infection in mice: hemagglutinin response and allograft survival. *J. Immunol.*, **106**, 364.

BLAIR, P.B. & MORETTI, R.L. (1970). Transplantation of isologous male mammary gland tissue into the mammary fatpads of female mice. *Transplantation*, **10**, 44.

DEN OTTER, W., RUNHAAR, B.A., RUITENBEEK, A. & DULLENS, H.F.J. (1974). Site-dependent differences in rejection of tumor cells with and without preimmunization. *Eur. J. Immunol.*, **4**, 444.

DEOME, K.B., FAULKIN, L.J. JR., BERN, H.A. & BLAIR, P.B. (1959). Development of mammary tumors from hyperplastic alveolar nodules transplanted into gland-free mammary fat periods of female C3H mice. *Cancer Res.*, **19**, 515.

DEOME, K.B., OSBORN, R.C., MIYAMOTO, M.J. & GUZMAN, R.C. (1980). A survey of mouse mammary tissues from eight strains and two hybrids for the presence of nodule-transformed cells. In: *Cell Biology of Breast Cancer*, (Ed. McGrath *et al.*), New York: Academic Press, p. 79.

DUNN, T.B. (1958). Morphology of mammary tumors in mice. In: *The Pathophysiology of Cancer*, (Ed. Homburger), New York: P.B. Hoeber, Inc., p. 38.

FAULKIN, L.J. JR. & DEOME, K.B. (1960). Regulation of growth and spacing of gland elements in the mammary fat pad of the C3H mouse. *J. Natl Cancer Inst.*, **24**, 953.

FIDLER, I.J. (1975). Biological behavior of malignant melanoma cells correlated to their survival *in vivo*. *Cancer Res.*, **35**, 218.

FIDLER, I.J., GERSTEN, D.J. & HART, I.R. (1978). The biology of cancer invasion and metastasis. *Adv. Cancer Res.*, **28**, 149.

FIDLER, I.J. & HART, I.R. (1981). Biological and experimental consequences of the zonal composition of solid tumors. *Cancer Res.*, **41**, 3266.

FOLKMAN, J. & HOCHBERG, M. (1973). Self regulation of growth in three dimensions. *J. Exp. Med.*, **138**, 745.

GIAVAZZI, R., SPREAFICO, F., GAVATTINI, S. & MANTOVANI, A. (1980). Metastasizing capacity of tumour cells from spontaneous metastases of transplanted murine tumours. *Br. J. Cancer*, **42**, 462.

HAGER, J.C., MILLER, F.R. & HEPPNER, G.H. (1978). Influence of serial transplantation on the immunological-clinical correlates of BALB/cfC3H mouse mammary tumors. *Cancer Res.*, **38**, 2492.

HEPPNER, G.H., DEXTER, D.L., DENUCCI, T., MILLER, F.R. & CALABRESI, P. (1978). Heterogeneity in drug sensitivity among tumor cell subpopulations of a single mammary tumor. *Cancer Res.*, **38**, 3758.

HEWITT, H.B. (1978). The choice of animal tumors for experimental studies of cancer therapy. *Adv. Cancer Res.*, **27**, 149.

KRIPKE, M.L., GRUYS, E. & FIDLER, I.J. (1978). Metastatic heterogeneity of cells from an ultraviolet light-induced murine fibrosarcoma of recent origin. *Cancer Res.*, **38**, 2962.

LIOTTA, L.A., ABE, S., ROBEY, P.G. & MARTIN, G.R. (1979). Preferential digestion of basement membrane collagen by an enzyme derived from a metastatic murine tumor. *Proc. Natl Acad. Sci.*, **76**, 2268.

LIOTTA, L.A., KLEINERMAN, J. & SAIDEL, G.M. (1974). Quantitative relationships of intravascular tumor cells, tumor vessels, and pulmonary metastases following tumor implantation. *Cancer Res.*, **34**, 997.

MILLER, F.R., MEDINA, D. & HEPPNER, G.H. (1981). Preferential growth of mammary tumors in intact mammary fatpads. *Cancer Res.*, **41**, 3863.

MILLER, F.R., MILLER, B.E. & HEPPNER, G.H. (1983). Characterization of metastatic heterogeneity among subpopulations of a single mouse mammary tumor: heterogeneity in phenotypic stability. *Inv. Metast.*, **3**, 22.

MORTON, D.L., MILLER, F.R. & WOOD, D.A. (1969). Demonstration of tumor-specific immunity against antigens unrelated to the mammary tumor virus in spontaneous mammary adenocarcinomas. *J. Natl Cancer Inst.*, **42**, 289.

PIESSENS, W.F. & CHURCHILL, W.H. (1977). Transplantable mammary tumors in Wistar/Furth rats: development, antigenicity, and effect of hormone manipulations. *J. Natl Cancer Inst.*, **59**, 911.

PITELKA, D.R., HAMAMOTO, S.T. & TAGGART, B.N. (1980). Epithelial cell junctions in primary and metastatic mammary tumors of mice. *Cancer Res.*, **40**, 1588.

POSTE, G. (1982). Experimental systems for analysis of the malignant phenotype. *Cancer Met. Rev.*, **1**, 141.

POSTE, G., DOLL, J., BROWN, A.E., TZENG, J. & ZEIDMAN, I. (1982). Comparison of the metastatic properties of B16 melanoma clones isolated from cultured cell lines, subcutaneous tumors, and individual lung metastases. *Cancer Res.*, **42**, 2770.

PREHN, R.T. (1970). Analysis of antigenic heterogeneity within individual 3-methylcholanthrene-induced mouse sarcomas. *J. Natl Cancer Inst.*, **45**, 1039.

PRICE, J.E., CARR, D., JONES, L.D., MESSER, P. & TARIN, D. (1982). Experimental analysis of factors affecting metastatic spread using naturally occurring tumours. *Inv. Metast.*, **2**, 77.

RYAN, J.N. & WOESSNER, J.F. JR. (1974). Oestradiol inhibition of collagenase role in uterine involution. *Nature*, **248**, 526.

SHELDON, W.G., OWEN, K., WEED, L. & KODELL, R. (1982). Distribution of mammary gland neoplasms and factors influencing metastases in hybrid mice. *Lab. Animal Sci.*, **32**, 166.

SMITH, H.S., RIGGS, J.L. & MOSESSON, M.W. (1979). Production of fibronectin by human epithelial cells in culture. *Cancer Res.*, **39**, 4138.

STACKPOLE, C.W. (1981). Distinct lung-colonizing and lung-metastasizing cell populations in B16 mouse melanoma. *Nature,* **289,** 798.

SWEENEY, F.L., POT-DEPRUN, J., POUPON, M.F. & CHOUROULINKOV, I. (1982). Heterogeneity of the growth and metastatic behavior of cloned cell lines derived from a primary rhabdomyosarcoma. *Cancer Res.,* **42,** 3776.

TARIN, D. & PRICE, J.E. (1981). Influence of microenvironment and vascular anatomy on "metastatic" colonization potential of mammary tumors. *Cancer Res.,* **41,** 3604.

TARIN, D. & PRICE, J.E. (1979). Metastatic colonization potential of primary tumour cells in mice. *Br. J. Cancer,* **39,** 740.

TOOLE, B.P., BISWAS, C. & GROSS, J. (1979). Hyaluronate and invasiveness of the rabbit V2 carcinoma. *Proc. Natl Acad. Sci.,* **76,** 6299.

VAAGE, J. (1978). Changing transplantation characteristics with serial *in vivo* passage of C3H/He mammary carcinomas. *Cancer Res.,* **38,** 3264.

VAAGE, J. & AGARWAL, S. (1976). Stimulation or inhibition of immune resistance against metastatic or local growth of a C3H mammary carcinoma. *Cancer Res.,* **36,** 1831.

WAHL, L.M. (1977). Hormonal regulation of macrophage collagenase activity. *Biochem. Biophys. Res. Comm.,* **74,** 838.

WEISS, D.W., FAULKIN, L.J. JR. & DEOME, K.B. (1964). Acquisition of heightened resistance and susceptibility to spontaneous mouse mammary carcinomas in the original host. *Cancer Res.,* **24,** 732.

WEISS, L. (1980). Metastasis: differences between cancer cells in primary and secondary tumors. *Pathobiol. Ann.,* **10,** 51.

WELCH, D.R., NERI, A. & NICOLSON, G.L. (1983). Comparison of "spontaneous" and "experimental" metastasis using rat 13762 mammary adenocarcinoma cell clones. *Inv. Metast.,* **3,** 65.

WEXLER, H., ORME, S.K. & KETCHAM, A.S. (1968). Biological behavior through successive transplant generations of transplantable tumors derived originally from primary chemically induced and spontaneous sources in mice. *J. Natl Cancer Inst.,* **40,** 513.

WHITE, A.C., LEVY, J.A. & McGRATH, C.M. (1982). Site-selective growth of a hormone-responsive human breast carcinoma in athymic nice. *Cancer Res.,* **42,** 906.

WICHA, M.S., LOWRIE, G., KOHN, E., BAGAVANDOSS, P. & MAHN, T. (1982). Extracellular matrix promotes mammary epithelial growth and differentiation *in vitro. Proc. Natl Acad. Sci.,* **79,** 3213.